# Editorial: Vascular smooth muscle cell fate and vascular remodeling: Mechanisms, therapeutic targets, and drugs, volume I

**DOI:** 10.3389/fphar.2022.989689

**Published:** 2022-08-16

**Authors:** Qilong Wang, Xiaoyan Dai, Vicky E. MacRae, Ping Song

**Affiliations:** ^1^ Institute of Traditional Chinese Medicine, Tianjin University of Traditional Chinese Medicine, Tianjin, China; ^2^ Guangzhou Municipal and Guangdong Provincial Key Laboratory of Molecular Target & Clinical Pharmacology, the NMPA and State Key Laboratory of Respiratory Disease, School of Pharmaceutical Sciences, Guangzhou Medical University, Guangzhou, China; ^3^ The Roslin Institute and R(D)VS, University of Edinburgh, Easter Bush, United Kingdom; ^4^ Center for Molecular and Translational Medicine, Georgia State University, Atlanta, GA, United States

**Keywords:** VSMC, neointimal hyperplasia, vascular dilation, arterial stiffness, calcification, atherosclerosis

## Introduction

Vascular smooth muscle cells (VSMCs), an essential cell type of the blood vessel, are required for maintaining vascular structure and function with unique phenotypes. However, under a pathological state VSMC fate changes such as proliferation, migration, apoptosis, quiescence, senescence and trans-differentiation, can lead to altered structure and arrangement of blood vessels. This can subsequently result in the development of critical cardiovascular diseases including atherosclerosis, aneurysm, hypertension, vascular calcification and arterial stiffness. Currently, there is limited therapy to prevent VSMC phenotype switching and vascular remodeling. Therefore, investigating the cellular and molecular basis of VSMC cell fate change will enable the discovery of novel therapeutic targets and develop effective medicines to treat cardiovascular diseases.

To understand the underlying mechanisms of VSMC fate regulation and issue future perspectives, we actively bring together this Research Topic “*Vascular Smooth Muscle Cell Fate and Vascular Remodeling: Mechanisms, Therapeutic Targets, and Drugs*” for the readers of Frontiers in Pharmacology. This Research Topic has twelve papers, including ten original research articles and two literature reviews, highlighting novel mechanisms and medicines underpinning VSMC fate and vascular remodeling.

## VSMCs and neointimal hyperplasia

Neointimal hyperplasia is a pathological process associated with dysregulated VSMC proliferation and migration within the vessel during atherosclerosis and in-stent restenosis. Zhang et al. showed that VSMC-specific deletion of lethal giant larvae 1 (LGL1), which functions as cell polarity regulator and tumor suppressor, caused promotion of neointimal hyperplasia *in vivo.* Moreover, LGL1 knockdown enhanced the proliferation and migration of VSMCs *in vitro*. The authors proposed that this effect may be mediated by the loss of LGL1-STAT3 binding and enhanced STAT3-mediated proliferation/migration-related gene transcription.

In-stent restenosis is a common complication following stent placement. Identifying the biomarker for the onset of in-stent restenosis in the patients is critical after stent implantation. Guo et al. (2022) recruited patients from 6 months and 2 years post percutaneous coronary intervention (PCI) and measured serum homocysteine. The authors observed a positive correlation between homocysteine and severity of restenosis after PCI, suggesting that serum homocysteine level might be a predictive biomarker for stent restenosis severity.

Additionally, new therapeutic medicines useful for suppressing neointima formation are illustrated here. Wu et al. (2022) found that theaflavin-3,3′-digallate, a natural product isolated from black tea, attenuated neointimal hyperplasia *in vivo*. Meanwhile, theaflavin-3,3′-digallate (TF3) decreased the proliferation and migration of primary rat aortic smooth cells *in vitro*. The authors further showed that TF3 reduced phosphorylation of PDGFRβ, leading to the blockage of PDGF-induced phenotypic switching of VSMCs, suggesting that TF3 might be a potential therapeutic candidate for the treatment of neointima formation.

## VSMCs and vascular dilation

VSMC contraction and relaxation contributes to the function of the vessel. However, abnormal vasoconstriction and vasospasm leads to vascular disease pathogenesis, particularly hypertension, angina and stroke. Zhang et al. found that benzoylaconitine, a monoester alkaloid from *Aconitum carmichaelii*, reduced blood pressure in spontaneously hypertensive rats. Studies demonstrated that benzoylaconitine directly binds with angiotensin-converting enzymes (ACE)/ACE2 and activates ACE/ACE2 activity, through virtual docking, surface plasmon resonance, enzyme activity assays and HUVEC cell culture experiments. Benzoylaconitine enhanced endothelium-dependent vasorelaxation and reduced vascular inflammation, and therefore maybe a potential modulator of the renin-angiotensin system for the treatment of hypertension.

Cui et al. (2022) found that allicin, an active molecular derived from garlic, exaggerated coronary artery relaxation induced by 5-hydroxytryptamine (5-HT), 9,11-dideoxy-9α,11α-methanoepoxy-prosta-5Z,13E-dien-1-oic acid (U46619), or endothelin-1 (ET-1). Allicin relaxed VSMCs via activation of the ATP-sensitive potassium (K_ATP_) channels. Moreover, Allicin enhanced hydrogen sulfide (H_2_S) production and cystathionine-γ-lyase levels in serum and myocardial tissue. These moleculesmay be involved in the mechanism of allicin action in acute myocardial infarction.

Traditional Chinese medicine has been used to treat cardiovascular disease for thousands of years. Guo et al. (2022) demonstrated that Danggui Buxue Decoction, consisting of *Angelicae Sinensis* Radix and *Astragali Radix*, induces a relaxation effect on rat middle cerebral artery. Danggui Buxue decoction, *Angelicae Sinensis* Radix, and *Astragali Radix* extracts relax KCl and U46619-contracted middle cerebral artery, with activation of K_ATP_ and K_ir_ channels underpinning this mechanism. Moreover, extracellular Ca^2+^ influx and internal Ca^2+^ from organelles also contribute to the action of Danggui Buxue Decoction. Fan et al. (2021) have further found that SaiLuoTong capsule attenuated cerebral infarction and neurological deficit in the middle cerebral artery occlusion rat model. SaiLuoTong capsule increased tight junction proteins via upregulation of a Nrf2-mediated anti-oxidative pathway in vascular endothelium and bone marrow microvascular endothelial cells, suggesting that SaiLuoTong capsule’s therapeutic effect on brain ischemia might be related to Nrf2-dependent endothelial cell protection.

## VSMCs and arterial stiffness

Arterial stiffness refers to the loss of elastic characteristics within the arterial wall, leading to systolic blood pressure and cardiac dysfunction. VSMC collagen deposition and hypercontraction contribute to arterial stiffness. Previous studies have measured the intrinsic mechanical properties of VSMCs to evaluate cell stiffness using atomic force microscopy. Ahmed et al. (2022) have provided a novel technique to record the tensegrity model of cellular mechanics using polyacrylamide hydrogels to mimic the physiological stiffness of the aortic wall. Angiotensin II inhibited the VSMC morphology and enhanced traction stress, whereas colchicine increased VSMC morphology, suggesting that VSMC morphology and actomyosin activity are the major reason for the contractile response. Moreover, microtubule destabilization by paclitaxel blocked the angiotensin II-induced morphology change, revealing that microtubules are essential in regulating the morphology and contractility of the isolated VSMCs.

Zhang et al. found that ginsenoside Rb1, a natural compound from ginseng, improved aortic stiffness in diabetic mice. Rb1 regulated pulse pressure and aortic compliance and restored acetylcholine-induced endothelium-dependent vaso-relaxation. Rb1 induced phosphorylation of AMPK and inhibited TGFβ1/smad2/3, ROS production, and MMP2/9 expression. Moreover, AMPK silencing blocked Rb1-mediated reduction of collagen deposition, fibronectin expression, and elastic fiber alignment, suggesting that Rb1 ameliorates diabetic arterial stiffness via AMPK activation.

## VSMCs and arterial calcification

Arterial calcification is characterized by the deposition of calcium phosphate crystals in the artery wall. VSMC transdifferentiation and mineralization can induce arterial calcification. Daprodustat is a medicine employed to increase erythropoiesis via stabilization of HIF1α. Toth et al. (2022) demonstrated that Daprodustat increased aortic calcification in a high phosphate-induced chronic kidney disease mice model. Daprodustat could stabilize HIF1α and HIF2α to accelerate medial calcification, suggesting that there is a possible risk that Daprodustat treatment could accelerate medial calcification in CKD patients with hyperphosphatemia.

## VSMCs and atherosclerosis

VSMCs are the primary source of plaque cells and extracellular matrix in both early- and late-stage atherosclerosis. Li et al. (2022) have reviewed the effect of extracellular vesicles on VSMC in atherosclerosis. The extracellular vesicles could be secreted by multiple cell types, including endothelial cells, macrophages, and mesenchymal stem cells. Extracellular vesicles are essential for intercellular communication via their contents, such as miRNA and lncRNA. The author suggested that extracellular vesicles might function as diagnostic indicators of atherosclerosis and drug vectors. Wang et al. (2022) have summarized the role of IL-17 in the pathogenesis of rheumatoid arthritis and atherosclerosis. Serum IL-17 level is significantly upregulated in patients with rheumatoid arthritis and atherosclerosis. Then, IL-17 regulates proliferation, migration, and apoptosis of vascular endothelial cells and VSMC, leading to cytokine production and the development of atherosclerosis. IL-17 also regulates bone destruction and synovial hyperplasia. Therefore, IL-17 might be used as a potential therapeutic target for the occurrence and development of cardiovascular disease in patients with rheumatoid arthritis.

In conclusion, this Research Topic provides valuable articles describing novel molecular mechanisms and innovative therapeutic medicines to treat cardiovascular diseases.
